# Immunotherapy for colorectal cancer

**DOI:** 10.3389/fimmu.2024.1433315

**Published:** 2024-08-21

**Authors:** Bing Yu, Jian Kang, Hong Lei, Zhe Li, Hao Yang, Meng Zhang

**Affiliations:** Department of the Colorectal Anal Surgery, The Affiliated Taian City Centeral Hospital of Qingdao University, Tai’an, Shandong, China

**Keywords:** colorectal cancer, immunotherapy, immune checkpoint inhibitors, adoptive cell therapy, oncolytic virus

## Abstract

Colorectal cancer is the third most common cancer and the second most lethal cancer in the world. The main cause of the disease is due to dietary and behavioral factors. The treatment of this complex disease is mainly based on traditional treatments, including surgery, radiotherapy, and chemotherapy. Due to its high prevalence and high morbidity, more effective treatments with fewer side effects are urgently needed. In recent years, immunotherapy has become a potential therapeutic alternative and one of the fastest-developing treatments. Immunotherapy inhibits tumor growth by activating or enhancing the immune system to recognize and attack cancer cells. This review presents the latest immunotherapies for immune checkpoint inhibitors, cell therapy, tumor-infiltrating lymphocytes, and oncolytic viruses. Some of these have shown promising results in clinical trials and are used in clinical treatment.

## 1 Introduction

Colorectal cancer (CRC) is the third most common malignancy in the world and the second leading cause of cancer deaths (approximately 1.9 million cases) and, thus, is one of the most serious public health problems in the world (1). Early diagnosis and treatment can achieve a satisfactory therapeutic effect. In contrast, survival is only 13.1% if diagnosed in the late metastatic stages. The incidence of CRC is increasing due to changes in the human lifestyle, such as reduced physical activity and increased consumption of high-fat foods (2). New cases of CRC will be more than 2.2 million worldwide, and 1.1 million deaths are estimated to occur by 2030, posing a serious threat to human health.

CRC is a genetically heterogeneous disease with different molecular pathways involved in tumor formation and metastasis (3). The development of CRC usually transforms from normal epithelial cells to uncontrolled proliferative epithelial cells that form polyps and carcinoma, respectively (4). Histologically, adenocarcinoma is the most common variant of CRC. Conventional treatment modalities include radiotherapy, chemotherapy, and surgical interventions (5). To prolong patient survival, the clinician usually adopts a variety of combined treatments depending on the location of the tumor and the mode of infiltration. For localized tumors, surgical treatment may be the best option. However, cancer cells are not completely removed, and residual cancer cells in local tissue, blood, and lymphatics usually lead to tumor recurrence. In recent years, immunotherapy has grown rapidly and relies on innate immunity and adaptive immunity, postoperative adjuvant chemotherapy, and immunotherapy to identify and remove residual cancer cells, with satisfactory therapeutic effects (6).

There are three main types of genetic instabilities in CRC, including chromosomal instability, CPG island methylation, and microsatellite instability (MSI) (7). The microsatellite refers to a class of short tandem repeat DNA sequences composed of 1~6 nucleotides in the genome, which are evenly distributed within the genome, rich in polymorphism information, and easy to detect (8). Normally, microsatellites are relatively conservative, also known as microsatellite stability (microsatellite stable, MSS). However, in disease states, such as tumors, the factors of double-stranded DNA replication can lead to the insertion or deletion of repeats, and replication errors form new microsatellite alleles (9). MSI is one of the most well-studied molecular markers in CRC. MSI indicates inactivation of the mismatch repair (MMR) gene and is usually associated with a CpG island methylation phenotype, while microsatellite stabilization (MSS) is associated with chromosomal instability (CIN). Approximately 15% of CRC patients presented had MSI, while the rest had MSS. MSI CRC also contains more point mutations than MSS CRC, but they have not been fully studied. Most mutations are for short nucleotide repeats, small insertions, and deletions (insertion deletions). Genes that confer cell growth advantage through loss-of-function mutations in microsatellites or MSI target genes have been extensively studied, and many have been published as candidate targets, thus being considered tumor suppressors. The heterogeneity of microsatellite status is widely used as a biomarker for the classification, treatment, and prognosis of genetic diseases and multiple tumors.

Immunotherapy is a therapeutic approach that activates and enhances the immune system to recognize and eliminate cancer cells to inhibit tumor growth (10). The immune system is composed of a variety of cells, tissues, and organs throughout the body to protect the body and remove pathogens, foreign bodies, and abnormal cells (11). Immunotherapy includes immune checkpoint inhibitors (ICIs), chimeric antigen receptor (CAR)-T cells, tumor-infiltrating lymphocytes (TILs), and oncolytic viral therapy (OVT) (12, 13). Studies have shown that melanoma, lung cancer, bladder cancer, and some types of blood cancer respond well to immunotherapy. This review introduces four immunotherapy methods for the treatment of CRC, explains their mechanisms, and discusses opportunities and challenges in the process of immunotherapy research. The aim is to let CRC patients understand the immunotherapy strategies, access the hope of cure, and expand the research ideas for the researchers.

## 2 Immunotherapy

### 2.1 Immune checkpoint inhibitors

Immune checkpoints are molecules expressed in T cells that inhibit T cells during the immune response and prevent the autoimmune response (14). Immune checkpoint inhibitors (ICIs) bind to receptors to disrupt immunosuppressive signals between antigen-presenting cells (APCs), tumor cells, and T cells, thus activating T cells, releasing killer factors, and killing tumor cells (15). CRC is highly heterogeneous at both the genetic and molecular levels, so treatment must be targeted to individual patients based on their unique molecular characteristics (16). Microsatellites are highly polymorphic repetitive DNA sequences in the human genome. The Cancer Genome Atlas (TCGA) project used comprehensive molecular analysis (chip-based sequencing technology) to classify CRC into two molecular pathological categories. These include microsatellite instability (MSI) and microsatellite stability (MSS) CRC (17). Approximately 13% of tumors are characterized by genomic instability of cancer cells due to lack of MMR. MSI CRC accounts for 15% of all sporadic CRC and can be divided into MSI-high (MSI-H) and MSI-low (MSI-L) based on the frequency of microsatellite marker instability (18). MSI-H typically has a sustained response to ICIs, including selective monoclonal antibodies against programmed cell death-1 (PD-1), programmed cell death ligand-1 (PD-L1), and cytotoxic T lymphocyte-associated antigen-4 (CTLA-4) (**Table 1**) (19). In contrast, approximately 85% of patients with CRC harboring microsatellite stable (MSS) tumors typically lack response to ICI. Currently, the most famous immune checkpoints of CRC immunotherapy include CTLA-4 and PD-1 (20, 21).

**Table 1:** FDA-approved monoclonal antibodies to treat colorectal cancer.

Name	Target	Data for launch	Types
Ipilimumab	CTLA-4	2011	For progressive microsatellite instability (MSI-H) or mismatch repair deficiency (dMMR) CRC (in conjunction with nivolumab)
Nivolumab	PD-1	2014	For progressive microsatellite instability (MSI-H) or mismatch repair deficiency (dMMR) CRC (in conjunction with ipilimumab)
Pembrolizumab	PD-1	2014	For unresectable or metastatic microsatellite instable-high (MSI-H) or mismatch repair-deficient (dMMR) CRC
Dostarlimab	PD-1	2021	For recurrent or advanced solid tumors with mismatch repair deficiency (dMMR)

**Figure 1:**
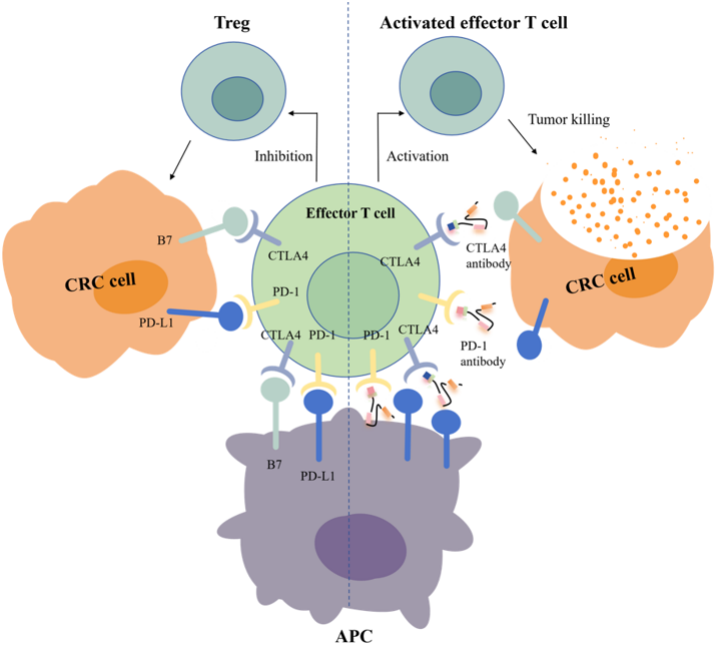
Mechanisms of action of PD-1 and CTLA-4 antibodies.

Another key target is CTLA-4, which is the first immune checkpoint to be discovered by James Allison in the 1990s. Studies showed that CTLA-4 can competitively bind to costimulator B7 with CD28, and the immunosuppressive effect after binding was significantly stronger than that of CD28 alone (**Figure 1**) (22). In 2011, ipilimumab, called Yervoy, which was a whole human monoclonal antibody against CTLA-4, was approved for the treatment of melanoma as the first CTLA-4 mab (23). Subsequently, researchers found that ipilimumab showed good therapeutic effect in metastatic colorectal cancer (mCRC) (24). However, in the course of clinical application, it was found that after CRC patients applied ipilimumab, there were severe immune-mediated adverse reactions, including fatigue, diarrhea, musculoskeletal pain, and rash. The therapeutic effect of ipilimumab attracts many researchers who are trying to reduce side effects by combining multiple drugs. A phase 2 clinical trial of 141 individuals showed that nivolumab combined with low-dose ipilimumab as first-line treatment for patients with high/mismatch repair deficiency (MSI-H/dMMR) (mCRC) demonstrated robust and durable clinical benefit with good tolerance (25). The results of the 4-year follow-up of the study also further confirmed the effectiveness of this combination treatment strategy (26).

Programmed cell death protein 1 (PD-1) is the most important receptor for activating T-cell expression and mediating immunosuppression, while programmed cell death ligand 1 (CD274, PD-L1) is involved in programmed death, leading to apoptosis or inactivation of T cells (27). A phase III trial showed that PD-1 blockade was associated with significantly longer progression-free survival and fewer treatment-related adverse events in MSI-H or dMMR CRC than chemotherapy (28). In 2002, evidence of PD-1 pathway-mediated tumor immunity was first reported: after PD-1 binds to PD-L1, tumor cells used recognition of T-cell receptors to further suppress immunity and evade immune surveillance, and then significantly promoted tumorigenesis and invasion (**Figure 1**) (29). PD-1/PD-L1 inhibitors can prevent T-cell apoptosis and dysfunction, thus further enhancing T-cell activation. However, only a small proportion of CRC patients with defective mismatch repair/high level of microsatellite instability (dMMR/MSI-H) showed a response to anti-PD-1/PD-L1 treatment. In 2014, the FDA approved two monoclonal antibodies PD-1 (nivolumab and pembrolizumab) for the treatment of dMMR/MSI-H CRC, showing good and stable therapeutic effects (12). The KEYNOTE-177 study comparing the efficacy of pembrolizumab with standard chemotherapy in the first-line treatment of dMMR/MSI-H mCRC showed that median progression-free survival (PFS) was 16.5 months (95% CI 5.4–38.1) with pembrolizumab and 8.2 months (6.1–10.2) (HR 0.59, 95% CI 0.45–0.79) with chemotherapy (30). Thirty-three of 153 patients (22%) with pembrolizumab treatment and 95 of 143 patients (66%) with chemotherapy had grade 3 or worse treatment-related adverse events. Compared with chemotherapy, pembrolizumab monotherapy was associated with longer PFS, higher objective and complete responses, and fewer treatment-related adverse events in patients with MSI-H/dMMR mCRC. Pembrolizumab or nivolumab alone or in combination with ipilimumab is recommended as a first-line treatment option in patients with dMMR/MSI-H mCRC in the 2021 National Comprehensive Cancer Network (NCCN) guidelines (31).

ICI therapy is an effective therapeutic strategy after surgical resection, chemotherapy, radiotherapy, and targeted therapy, with great potential in the treatment of CRC. ICIs have fundamentally altered the prognosis of MSI CRC patients. In an open-label phase III study (KEYNOTE-177), 83% of pembrolizumab-treated patients with metastatic MSI-H-dMMR CRC had a sustained response at 24 months compared to 35% in the chemotherapy group. Despite these advances, MSI-H-dMMR CRC represents only a small subset of CRC, and ICI is largely ineffective in metastatic MSS-pMMR CRC, which represents the majority of patients (32). A phase II CheckMate 142 study reported an objective response rate of 69% with nivolumab combined with low-dose ipilimumab, and its effectiveness warrants first-line dual ICI therapy in a randomized study (25). At the 2024 American Society of Clinical Oncology (ASCO) Digestive Oncology Symposium, the research institute announced the study of CheckMate-8HW (NCT04008030). CheckMate-8HW is a randomized, open-label phase III clinical trial that evaluates the efficacy of nivolumab + ipilimumab versus nivolumab monotherapy or chemotherapy (mFOLFOX-6 or FOLFIRI) with or without bevacizumab/cetuximab in patients with mCRC with high microsatellite instability (MSI-H) or mismatch repair-deficient (dMMR) phenotypes. The results showed that nivolumab + ipilimumab reduced the risk of disease progression or death by 79% in patients with MSI-high or mismatch repair-deficient metastatic colorectal cancer. This study helps define the additional benefit of nivolumab plus ipilimumab versus nivolumab alone and helps clinicians determine the best treatment for their patients.

The FDA has approved multiple monoclonal antibodies for the treatment of CRC, including cetuximab, bevacizumab, panitumumab, ramocumab, ipilimumab, and pembrolizumab, which respond well to cancer. **Supplementary Table 1** shows the clinical trials completed with ICI for CRC. These include several combination treatment strategies that have shown great promise in improving the overall clinical outcome of patients. Despite some progress, challenges remain in the widespread use of monoclonal antibody (mAb) in CRC therapy. First, the status of MMR/MSI, RAS, and BRAF before CRC treatment and the detection of the mutation status are important to guide clinical medication, develop personalized medication regimens, and benefit more patients. Second, as an emerging treatment method with great potential, its safety profile cannot be ignored. The question of how to reduce the side effects of treatment and reduce patients’ pain is one of the research emphases. Therefore, clinicians try to combine several different immunotherapy drugs to reduce the concentration and side effects of a single drug. Third, radiotherapy, chemotherapy, and ICI, combined with immunotherapy in CRC, are utilized to achieve the best effect and the least side effects. Issues such as patient selection, biomarker identification, and resistance mechanisms must be addressed to optimize the use of mAbs in clinical practice. In conclusion, ICI can change the therapeutic prospects of CRC, allowing more patients to benefit from treatment.

### 2.2 Adoptive cell therapy

Cellular immunotherapy for cancer is also known as adoptive cell therapy (ACT). It is a type of immunotherapy in which the cells of the body’s own immune system are genetically modified to express a CAR or a T-cell receptor (TCR) to eliminate cancer (33). ACT has played an important role in the treatment of many types of tumors. ACT offers several advantages over other cancer immunotherapies. Large numbers of antitumor T cells can be grown *in vitro*, and their antigen affinity can enhance autoimmunity (34). Following the gradual deepening of the TIL research, cells with antitumor activity were isolated from the tumors of patients with melanoma and showed good therapeutic effects. Despite the use of similar techniques, TILs grown from most CRC tissues do not appear to recognize tumor antigens. Further application of ACT led to the development of techniques to introduce antitumor TCRs into autologous lymphocytes for therapeutic use (35). CARs with antitumor specificity can be introduced into normal lymphocytes to enhance antitumor activity (36). CAR T-cell therapy uses gene transfer technology to reprogram a patient’s own T cells to stably express CAR, thereby combining antibody specificity with the potent cytotoxic and memory capabilities of T cells (37). In early-phase clinical trials, CD19-targeted CAR T cells produced complete sustained remissions in populations of patients with refractory B-cell malignancies and, more specifically, showed complete response rates of approximately 90% in patients with relapsed or refractory acute lymphoblastic leukemia.

#### 2.2.1 CAR T cell

**Figure 2:**
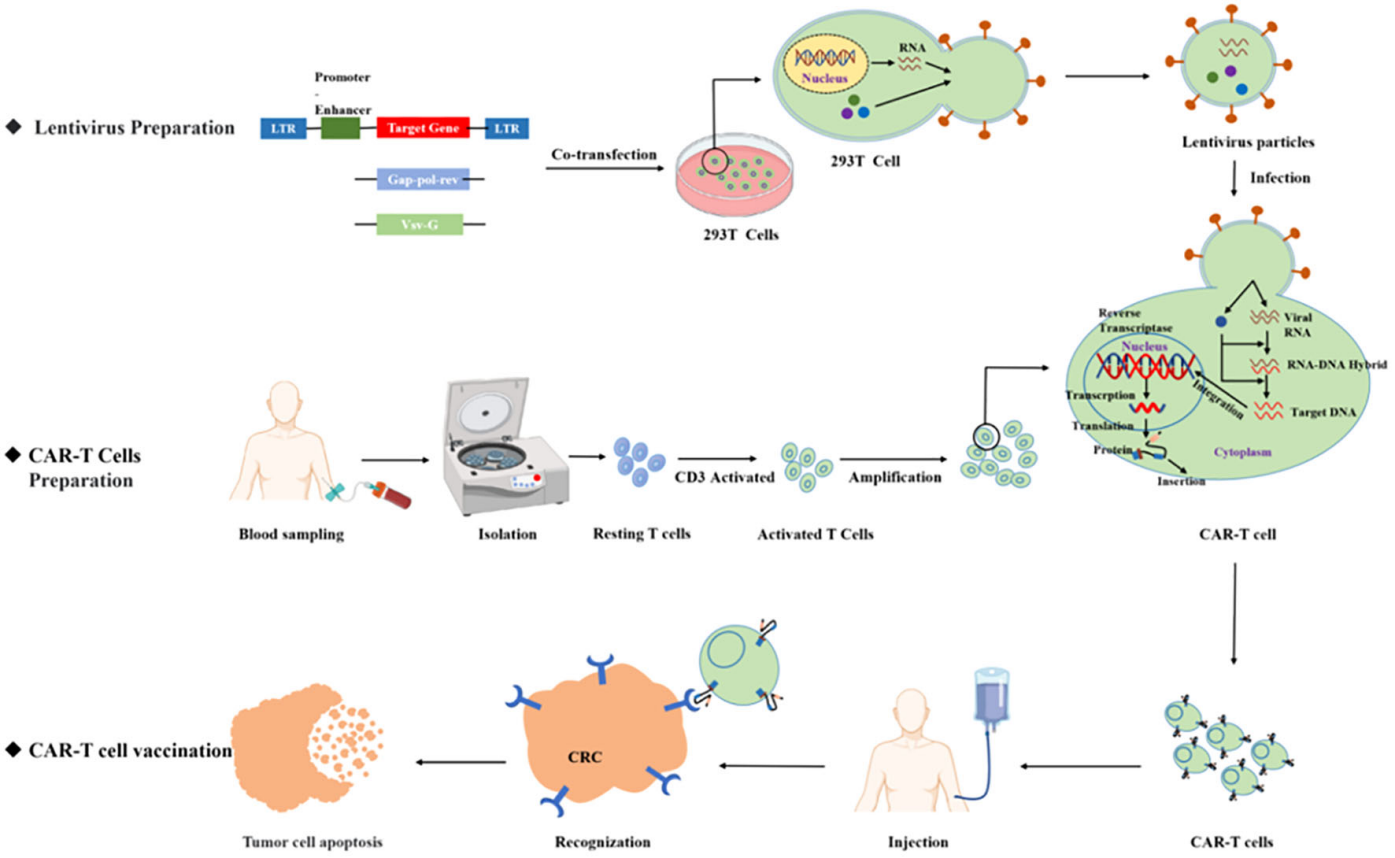
CAR T product preparation process.

**Figure 3:**
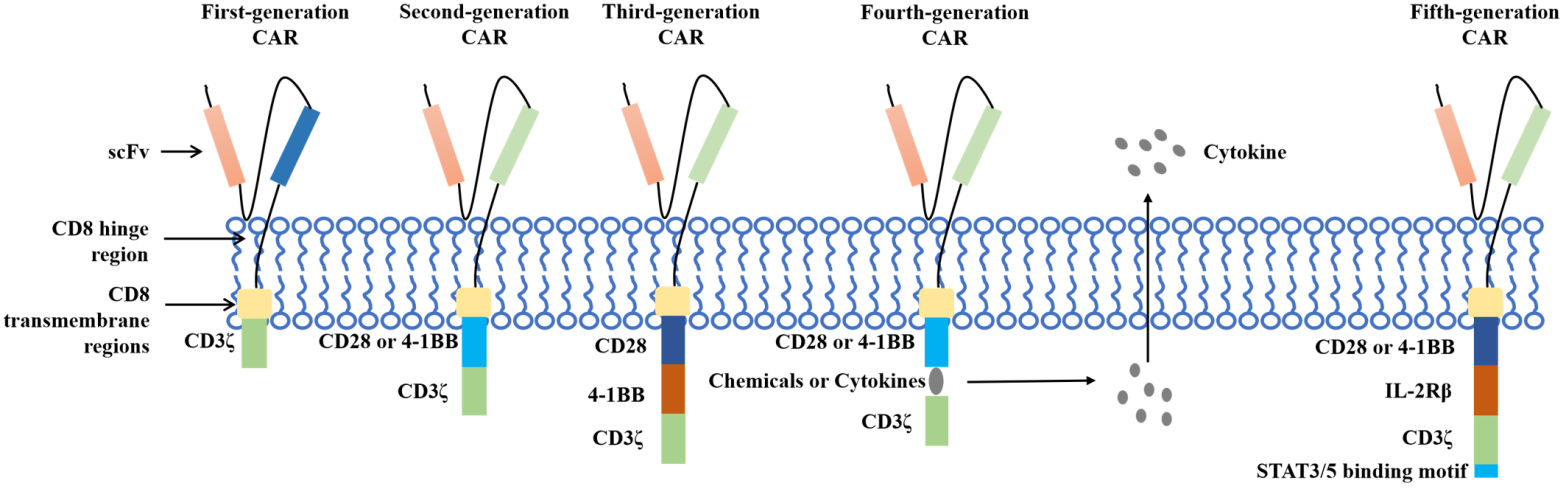
The development of CAR T.

Chimeric antigen receptor T (CAR T)-cell therapy is an important breakthrough therapeutic tool for cancer research and is a personalized therapy that has achieved great success in the treatment of hematological malignancies. CAR T therapy isolates patient lymphocytes from peripheral blood and genetically modifies autologous T cells. Lentiviral vectors or retroviruses are used to modify T cells *in vitro* by genetic engineering technology to produce specific receptors for tumor antigens. Modified T cells can identify cancerous antigens independently of MHC and produce a specific antitumor immune response (**Figure 2**) (38). CAR T cells express synthetic receptors and redirect to the tumor surface antigen, release perforin, and granulin B to kill tumor cells directly. Through the release of cytokines, endogenous immune cells kill tumor cells and achieve the purpose of treating a tumor. CAR T cells can form immune memory T cells to obtain a specific long-term antitumor activity (39). In recent years, CAR T technology has developed rapidly, and innovative manufacturing processes and transformation strategies have gradually increased the stability and effectiveness of CAR T and have reduced costs and side effects. The CAR comprises a target-binding extracellular region with antigen specificity usually based on antibody fragments from a single-chain variable region (scFv), hinge regions, transmembrane regions, and an intracellular domain that mediates T-cell activation, mainly via the CD3 ζ signaling chain (40). Since the first generation, CAR T-cell production has been optimized to the fifth generation, six CAR T-cell products have been approved worldwide, and countless products are in preclinical and clinical trials. The fifth generation of CAR T tries to break through the limitations of individuals and is used for large-scale production and treatment (**Figure 3**) (41, 42).

CAR T-cell immunotherapy is a very promising anticancer strategy that has attracted many researchers to explore modification strategies for CRC. Several preclinical and clinical studies are still in the primary stage (phase I/II clinical trials) to evaluate the efficacy, safety, dose levels, and maximum tolerated dose of CAR T cells against various overexpressed molecular targets in CRC (**Supplementary Table 2**). ClinicalTrials.gov showed that the targets used most frequently in CAR T-cell therapy in CRC studies were CEA and NKG2DL, followed by EGFR and HER-2. In 2017, a phase I increase in the doses of CAR T therapy targeting CEA mCRC was conducted. The results indicated that two patients had stable disease for more than 30 weeks and two patients had significant tumor reduction (43). Whether these findings reveal a good treatment effect and can be applicable to all patients remains to be verified by further clinical trials.

With the development of cell technology, researchers no longer focused on T cells and began to test other CAR immune cells and even the combination of multiple immune methods. In 2019, researchers fused the extracellular domain of cell receptor NKG2D of natural killer (NK) cells with DAP12 to enhance NK cell tumor response. The preclinical trial showed good therapeutic effects in CRC tumor-bearing mice, and a preliminary clinical trial (NCT03415100) was conducted (44). Preliminary verification showed that NKG2D CAR-NK cells can identify tumor cells and exhibit antitumor effector functions in patients with mCRC. In 2020, researchers constructed CYAD-101, using a non-gene-edited peptide-based technology (TIM) in combination with NKG2D-based CAR to control graft-versus-host disease (GvHD) (45). Preclinical findings showed that CYAD-101 still maintained the CAR-directed antitumor activity in the absence of induced GvHD. Subsequently, a phase I alloSHRINK clinical study (NCT03692429) enrolled 15 patients with refractory mCRC who had previously failed at least first-line treatment with oxaliplatin, showing 2 patients in partial remission and 9 with stable results. This study demonstrated the attempt of allogeneic CAR T-cell therapies to overcome the limitations of autologous CAR T and was presented at the American Society of Clinical Oncology Gastrointestinal Cancer Symposium 2021 (ASCO-GI). In 2023, researchers secreted bispecific PD-1-TREM2 scFv antibodies into the tumor microenvironment (TME) which could simultaneously target PD-1, TAM, and MDSC. In the CRC mouse model, CAR T cells for BsAb PD-1-TREM2 scFv secretion were shown to exhibit a stronger antitumor potential (46). This study innovatively combined CAR T cells and BsAb into a single immunotherapy platform with greater antitumor efficacy in tumor-bearing mice, prompting new research for the study of CAR T. Despite the impressive success of CAR T-cell therapy in hematological malignancies, particularly CD19-positive B-cell malignancies, the development of CAR T-cell therapy in solid tumors has stalled (47). Ongoing efforts are advancing basic research in this field, with several studies progressing to clinical trials and multiple combination strategies proposed to further improve efficacy and safety (**Supplementary Table 2**). Each strategy has a mechanism of action and various advantages or limitations. A variety of targets are available for CAR treatment of CRC, and many promising therapeutic strategies have been proposed and shown to be successful in preclinical models. The important role of CAR therapy in solid tumors has been demonstrated in various studies (48). In addition to engineering with T cells, NK cells have been considered an alternative vehicle for CAR constructs and are thought to be less susceptible to GvHD. CAR-NK cells can expand the therapeutic scope of solid tumors and extend to allogeneic CAR therapy (49). CAR-related toxicity often presents acutely, and control mechanisms should ideally allow the rapid control of CAR T-cell activity (50).

CAR T cells are one of the most studied and promising methods among ACTs, and although clinical trials are still in the early stages, the results have shown promising therapeutic effects. Some barriers and limitations must be addressed during the study. First, the TME is an important limitation of CAR T-cell therapy. The TME presents local tissue hypoxia, nutrient metabolism disorders, and more acid products from hypermetabolism. These factors affect T-cell survival, proliferation, and activation and also limit the inhibitory effect of CAR T cells on tumors. Whether personalized modification of CAR T cells, such as integration with antitumor cytokines, inoculation mode, and dose control, can reduce the impact of the TME still needs further exploration. Second, CAR T-cell therapy can cause many toxic effects. One of the most common is the cytokine release syndrome (CRS), which is the CAR T-cell infusion cytokine secretion reaction and causes other systemic toxicity. Patients may have respiratory circulation disorders, liver dysfunction, gastrointestinal reaction, and neurotoxic reaction, even in a short period of time, which can be life-threatening. Researchers are also constantly trying to minimize side effects and benefit more CRC patients. The selection of CAR T cells should also be adapted to the type of target tumor, as tissue-specific vascularization can prevent adequate biodistribution, concentration, and persistence of CAR T cells in affected organs (51). Positive results from clinical trials are now expected to provide hope for this emerging cell-based therapy.

#### 2.2.2 Tumor-infiltrating lymphocytes

TILs are an ACT which extract immune cells from tumor tissue, enhance their antitumor vitality *in vitro*, and are reinjected into the TME to enhance the immune activity to inhibit tumor growth (52). TILs generally represent a heterogeneous population of αβ T cells present in the TME, consisting of CD4^+^ and CD8^+^ subpopulations. These cells differentiate into killer cells that release perforin and express the apoptosis inducer FASL after amplification. Perforin disrupts the cell membrane and helps the granzyme enter the cell. Then, caspase precursors will undergo cleavage and induce apoptosis of the tumor cells (53). Since the distributions of TILs are different in different types of TME, it is particularly important to choose which kind of TILs. Researchers are trying to explore the relationship between TILs and rehabilitation to screen for TILs for cancer treatment. However, the production and responsiveness of TIL products to solid tumors vary due to individual differences (54, 55). Studies have suggested that TILs are an important prognostic factor in infiltrating or peripheral CRC (56, 57). Studies have shown that CD8^+^ T-cell infiltration was consistently higher than infiltration of CD4^+^ T cells in the CRC TME and was associated with a better prognosis in CRC. CD8^+^ TILs mediate the tumor rejection response by recognizing the tumor-associated antigens (TAAs) and directly killing transformed cells (58). Effector CD8 T cells in the TME produce IL-2, IL-12, and IFN-γ to enhance the cytotoxic potential of CD8 TILs to target the tumor cells (59).

**Figure 4:**
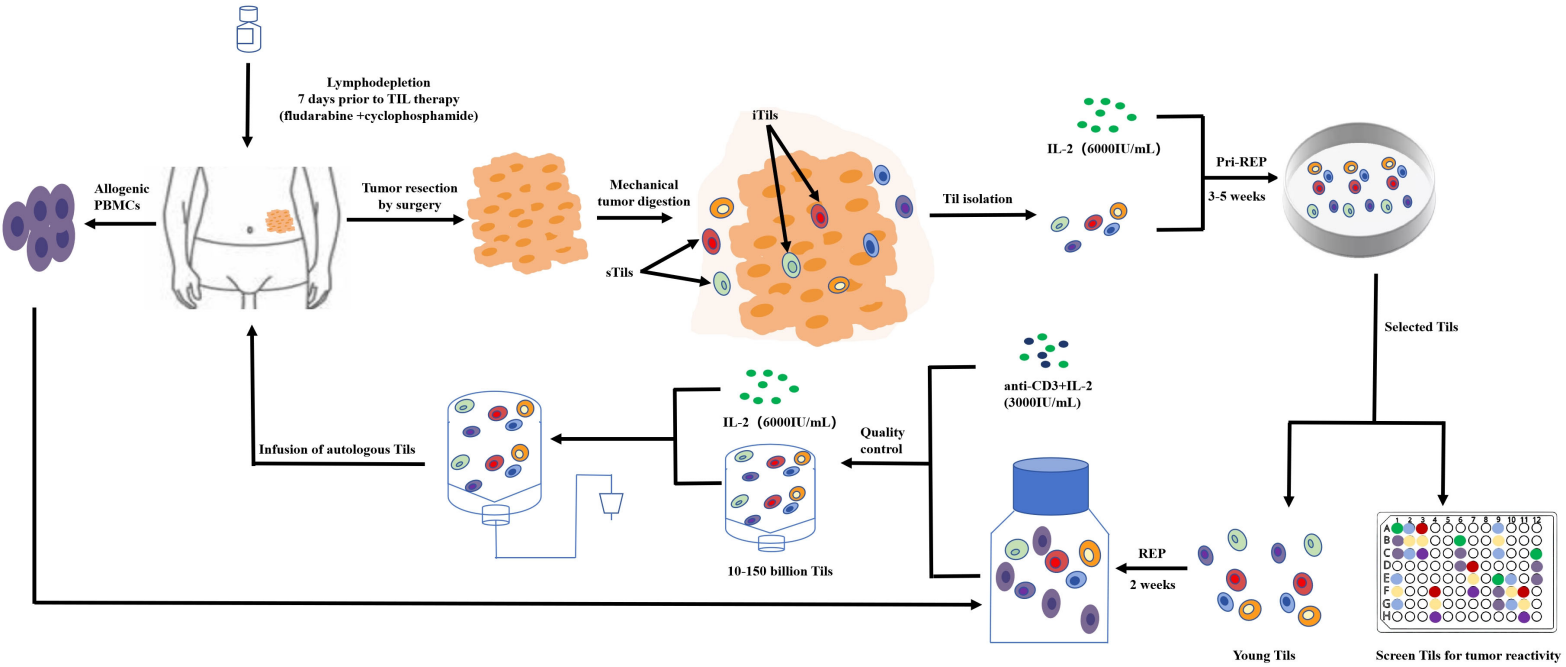
TIL product preparation process.

Fewer TILs are present in CRC than in other kinds of cancers; therefore, collecting and increasing the amount of treatment of TILs is one of the challenges of TILs for the treatment of CRC (60). Currently, researchers are using the traditional collection and *in-vitro* expansion methods for the rapid expansion protocol (REP) of the T cell. Under anesthesia, tumors are excised from the patient and are cut into small pieces or digested enzymatically to obtain a single-cell suspension. Tumor fragments are then cultured alone in the presence of high-dose IL-2 (6,000 IU/mL) for 3–5 weeks prior to REP. The selected pure lymphocyte cultures will then be co-cultured with lymphocytes and tumor cells in the presence of irradiated feeder lymphocytes (an antibody targeting ϵ subunits within human CD3) and IL-2 to rapidly expand for 5 to 6 weeks. The patient will then be transfused with cells (**Figure 4**).

Researchers have successfully designed an *in-vitro* amplification model for TILs (61). Tumor tissues from 12 patients undergoing surgery for primary CRC surgery were cut for pathological examination and transferred to a one-time perfusion bioreactor with a starting medium containing IL-2 and IL-12. The expanded TILs consisted mainly of (73%) the ACT-relevant CD3^+^/CD8^+^ effector memory phenotype (CD45RO^+^/CCR7), confirming that the amplified TILs showed high functional potential by measuring non-specific stimulation (interferon-γ, tumor necrosis factor-α cytokine assay), taking an important step in the immunotherapy of TILs.

How to expand effector T cells more efficiently and make them exert their antitumor effect is also one of the difficulties to overcome in TIL therapy. Preclinical studies have shown that CD8-dominated TIL produced a stronger antitumor capacity when using anti-4-1BB and CD3 antibody agonists in early isolated TIL culture (62). This single-center TIL was treated for a phase II trial (NCT03610490) for patients with CRC, PDAC, and OVCA refractory to standard therapy. The results showed that the DCR is 62.5% but the ORR is 0% and the median PFS and OS were 2.53 months and 18.86 months, respectively. The single-arm study was unable to conclude the efficacy of TIL compared to standard second- or third-line treatment options in different cohorts. However, the results of this experiment showed the effect of TIL therapy in inhibiting solid tumors. More studies are needed to identify host factors associated with resistance to TIL therapy.

TILs play an important role in identifying and killing target tumor cells and have achieved good results in recent years. However, there are still many challenges in the development process, such as treatment safety, a long production cycle, high production costs, optimization of manufacturing processes, and the use of innovative genetic modification techniques to create more effective TIL cell therapies. Several TIL/CAR T-cell trials have been associated with safety concerns, particularly the development of adverse effects on and off the target, including CAR T-cell-associated encephalopathy syndrome (CRES), extratumoral effects, and acute respiratory distress syndrome due to targeted humoral recognition and killing (63). Furthermore, CRS is the most common side effect of CAR T therapy (64). Although timely pharmacologic intervention is effective in managing most adverse events, ACT can persist for a long time, accompanied by any adverse effects (65). Conversely, tumor-restricted expression of neoantigens driven by somatic mutations ensures the therapeutic generation of cellular therapeutic reactivity against these antigens, which is independent of normal tissue toxicity and is considered an ideal and safe solution for ACT. However, with continuous improvement and advancement of technology, it is likely that effective TILs will be designed in the future to bring hope to CRC patients.

### 2.3 Oncolytic virotherapy

**Figure 5:**
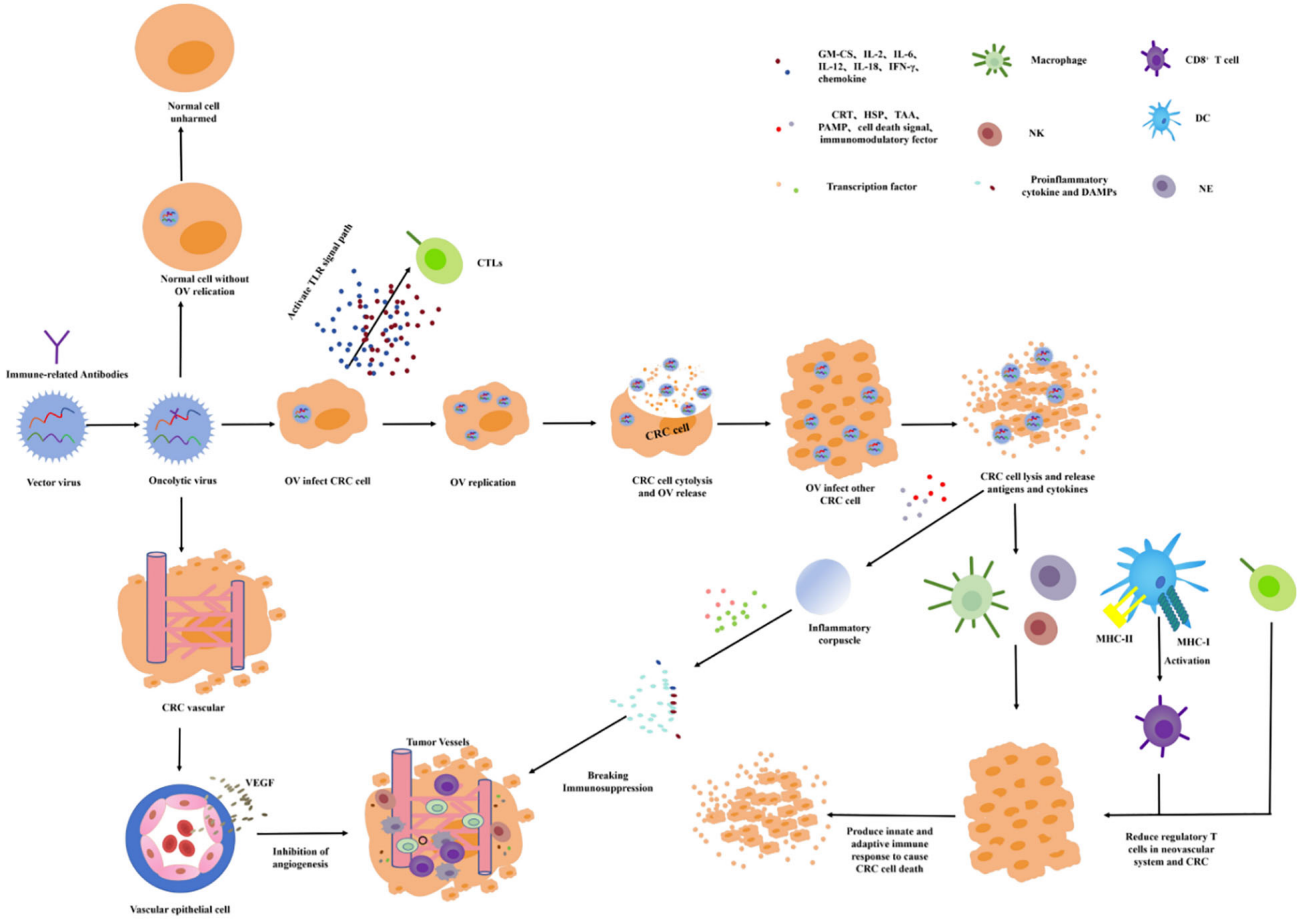
Mechanisms of oncolytic virus action.

OVT chooses a small virus as the viral vector and chimeric antitumor genes and immune factors to increase the targeting and immune activity. Oncolytic viruses (OVs) can infect the tumor cells by intratumoral administration, intravenous administration, and cell carrier delivery to target proliferation in the tumor cells. In the process of value-added process, the immune factors or tumor-related antibodies are released to directly induce oncolysis, activate the immune response, and drive immune cells toward the TME to inhibit tumor growth (**Figure 5**) (66, 67). The release of antiviral cytokines (especially interferons) is used to initiate antiviral responses, which promote the maturation of APCs such as dendritic cells (DC) and stimulate CD8 T cells and NK cells. The lysis of the infected tumor cells will release the viral progeny, DAMP (including host cell proteins), PAMP (viral particles), and TAAs into the TME (68, 69). The virus offspring will infect additional nearby or distal tumor cells. DAMPs and PAMPs stimulate the immune system by activating receptors. TAAs and neoantigens are absorbed by APCs to activate antigen and virus-specific CD8 T-cell responses and, finally, to create an immunostimulatory environment (70). If the OVs are chimeric with VEGF antibodies, VEGF antibodies will be released into the TME, and the VEGF function will be inhibited. Therefore, tumor-nourishing vessels will be inhibited and local blood perfusion will be reduced, resulting in tumor cells lacking oxygen and nutrients needed for growth.

OVT is one of the most promising immunotherapies for CRC. In recent years, OVT has achieved satisfactory results at both the cellular and organismal levels, and the research direction is increasingly turning to clinical trials. Currently, the most commonly used viruses for OVT research are the poxvirus, reovirus, herpes simplex virus (HSV), and adenovirus. Some tumor suppressor genes or antibodies that enhance immune response are transformed into viral DNA to play an oncolytic role in the amplification or metabolism of OVs (71). In the study of OVs for the treatment of CRC, researchers have tried to use OV technology to target the human 5T4 gene, CEA, PD-1, and CTLA-4 for the treatment of CRC. Modification of OVs has achieved a good therapeutic effect in preclinical research and is expected to provide a new treatment strategy for the clinical treatment of CRC (72).

The human 5T4 gene is known as the trophoblast glycoprotein. It is located at 6q14.1 and expresses a heavy N-glycosylated protein of 72 kDa. 5T4 is highly expressed in human trophoblast cells and most tumors but is rarely expressed in normal tissues (73). Researchers modified vaccinia Ankara-5T4 to treat inoperable mCRC with chemotherapy. This randomized phase I and phase II clinical trial in patients evaluated the effectiveness of cyclophosphamide in increasing the therapeutic potential of modified vaccinia Ankara-5T4 immunotherapy. The enrolled patients showed better tumor control effects when given cyclophosphamide or MVA-5T4 (74). Although cyclophosphamide failed to enhance the immunogenicity of MVA-5T4, its survival benefit and minimal adverse effects have been demonstrated, thus requiring further investigation.

The CEA subgroup is expressed mainly on the cell membrane. For more than 50 years, CEA has been identified as an important marker of CRC and other malignancies. The CEA gene promoter constructs can strongly inhibit CEA-producing adenocarcinoma cells (75). The insertion of the ST13 tumor suppressor gene and the CEA promoter E1A (Δ24) into an oncolytic adenovirus vector inhibits the growth of the SW620 CRC xenograft in nude mice and prolongs the survival time of mice (76). A team of researchers has already filed a patent protecting the invention (201110319434.4). Researchers constructed a recombinant oncolytic herpes simplex virus type 1 (HSV-1), called VG2025, which uses the dual regulation of transcription and translation (TTDR) of key viral genes to improve viral safety and promote tumor-specific viral replication without reducing virulence (77). VG2025 can efficiently replicate virally in CEA-positive cancer cells, promoting oncolysis and the release of tumor antigens while limiting viral replication in healthy tissues. The CEA promoter in VG2025 can be replaced by other tumor-specific promoters as part of a broader platform to facilitate biomarker-based precision OVT.

OVT has produced promising results for CRC in clinical and preclinical studies. With advances in molecular techniques, the safety and specificity of OVs have been achieved. However, many challenges still hampered the optimal antitumor activity of OVs. How are oncolytic viruses engineered more effectively? The host’s antiviral immunity needs to be taken into consideration, and how to successfully deliver the OVs remains the biggest challenge (78). Currently, the combination of OVs and ICIs has shown promise in multiple clinical trials. This strategy is expected to be a promising therapeutic option for CRC.

## 3 Discussion

Immunotherapy is a therapeutic approach that utilizes various cytokines, antibody drugs, OVs, and immune cells to activate or enhance the immune system to inhibit tumor growth (79). Since the FDA approved the first mAb, more than 100 mAb products have been approved with significant therapeutic efficacy (80). The ultimate goal of different tumor immunotherapies is to improve the precision of targeted therapies and reduce adverse effects while effectively eliminating tumor cells. This review describes the development of ICIs, cell therapy, TILs, and OV therapy in CRC. The mechanisms of several immunotherapies are described and ongoing immunotherapy research is introduced for patients with CRC to provide hope for treatment and to provide new research ideas for researchers.

High infiltration of specific subsets of immune cells in the immune microenvironment of type I CRC has been associated with improved survival and reduced risk of recurrence in patients with stage I/II CRC (81). However, patients with MSS CRC have been reported to have a high mortality risk in multiple cohorts. MSI/MSS subtyping has changed the diagnosis and treatment strategy of CRC. Indeed, patients with MSI-H are not sensitive to fluorouracil treatment; therefore, the combination chemotherapy regimen (FOLFOX) [(fluorouracil, 5-FU), oxaliplatin (oxaliplatin), and folic acid (folinic acid)] is less effective (up to 73.6% insensitive) than in patients with MSS CRC (only 26.6% insensitive) (82). Therefore, it appears that clarifying the microsatellite status of patients before CRC treatment is extremely valuable to guide treatment stratification.

The practical landscape for immunotherapeutic agents in solid tumors of MSI-H is evolving rapidly. Different strategies are under investigation, but the vast majority of them include combinations of anti-*PD(L)-1* agents with other immunomodulators. Anti-CTLA-4 plus anti-PD(L)-1 is the only combination to date that has shown better survival in patients with MSI-H cancers (25). The optimal timing of immunotherapy intervention, the duration of immune agents, the appropriate dose of immune drugs, and the combination strategy of preoperative immunotherapy and the cytotoxic profiles of these drugs are unknown (83). A short-course radiotherapy treatment paradigm before CRC surgery followed by 4–5 cycles of anti-PD1 therapy combined with fluorouracil or its derivative chemotherapy appears to be more ideal (84). Screening for patients who may benefit from immunotherapy is still based on MSI/MMR status: while some patients with MSI-H/dMMR CRC do not benefit from ICI treatment, other patients with MSS/pMMR can achieve a good clinical response from immunotherapy (85, 86). With the breakthrough of anti-PD-1 therapies, such as pembrolizumab and nivolumab, targeting the MSI-H:dMMR patient population, we have begun to see the potential of immunotherapy (87). However, in MSI-H tumors, there is also an urgent need to identify new biomarkers to predict the benefit of immunotherapy and possibly the benefit of specific immunotherapy agents. With the advent of cutting-edge technologies such as scRNA-seq, high-parameter flow cytometry, and spatial transcriptomics, our understanding of antitumor immune responses has been revolutionized. As these techniques become more advanced and widely adopted in the future, they have the potential to provide us with important insights into the behavior of immune cells in the TME. This knowledge can be used as a basis for designing more effective immunotherapies.

Tumor immunotherapy has become the main development direction and the trend of development of tumor therapy and is expected to become the final method of tumor treatment. However, the efficacy of these immunotherapies is limited by multiple mechanisms, including the emergence of compensatory inhibitory mechanisms that negatively regulate the antitumor immune response, leading to acquired resistance. The search for new tumor targets, the study of signaling mechanisms, and the development of new technologies are constantly increasing. Due to their novel, complex, and technical nature, these therapies can pose previously untapped risks to public health and individual patients. ICI therapy, TIL cell therapy, CAR T therapy, and OV vaccines were considered to have low to moderate risk. Potential risk factors include bioactive substances used in manufacturing, such as antibodies, cytokines, sera, growth factors, and antibiotics, as well as risks to the stability and viability of the product during storage, freezing, thawing, and cold chain transport. In addition, the product itself can carry inherent risks, such as incomplete removal of tumor cells or other unwanted cells, and potential complications or reduced product activity associated with homing, transplantation, migration, and proliferation. Researchers have tried to combine several drugs to reduce the concentration of each single drug and to reduce adverse drug reactions and have also achieved good experimental results. Combination therapy has shown superiority in some studies. In addition to different combinations of immunotherapy, immunotherapy combined with chemotherapy, radiotherapy, or other drugs has been explored, with a focus on improving efficacy, reversing resistance, and reducing adverse effects. In conclusion, immunotherapy is currently the most promising treatment modality and is expected to be a new therapeutic strategy for patients with CRC.
